# The Impact of Being Born and Growing Up Postliver Transplantation, Looking Back 35 y Later

**DOI:** 10.1097/TP.0000000000005078

**Published:** 2024-07-23

**Authors:** Jildau R. Meinderts, Adelita V. Ranchor, Margriet F.C. de Jong

**Affiliations:** 1 Department of Nephrology, Groningen Institute for Organ Transplantation, University Medical Center Groningen, University of Groningen, Groningen, the Netherlands.; 2 Department of Health Psychology, University Medical Center Groningen, University of Groningen, Groningen, the Netherlands.

Studies have shown that pregnancy after liver transplantation (LT) has increased risks of pregnancy complications but leads in the vast majority to healthy newborns. However, these studies have mostly focused on short-term outcomes.^1-3^ Little knowledge exists on long-term follow-up of mother, child, and the quality of life of these families. Here, we present a case report of what is, to our best knowledge, the first pregnancy after LT in Europe. In this case report, both mother and child are in good health 35-y postpregnancy. However, during individual interviews with mother, father, and child, we noticed that the quality of life, especially the child, is impacted during the entire childhood, which is an important aspect of pregnancy after transplantation that might be overlooked and warrants further investigation.

## CASE REPORT

**FIGURE 1.**
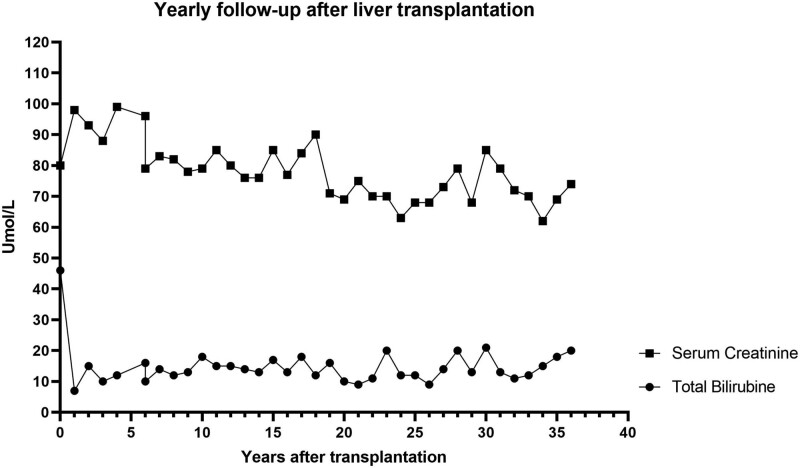
Yearly follow-up of serum creatinine and bilirubin levels after liver transplantation.

In 1987, the 26-y-old patient underwent LT receiving a graft from a brain-dead donor. The patient’s health has remained relatively stable, with Figure 1 illustrating a favorable 36-y follow-up of serum creatinine and bilirubin. Despite biliary cirrhosis, there has been no decompensation or graft rejection, and kidney function only mildly declined (last Chronic Kidney Disease Epidemiology Collaboration estimated glomerular filtration rate 74 mL/min × 1.73 m^2^).

The patient unexpectedly discovered her pregnancy a few months posttransplant. At 37 wk, a healthy girl weighing 2250 g was born via spontaneous vaginal delivery. Reflecting on the pregnancy 35 y later, the patient views it as a joyful period of *living in a bubble.* The father, however, experienced worries and anxiety. Up until now, no health problems have occurred in the daughter. However, although the parents felt *like any other family*, the daughter recalls multiple birthdays that needed to be celebrated in the hospital because of admission of her mother. “So yes, there’s always a kind of fear of, that you think, how, when is this really going to go wrong? I’ve been on the verge of losing her a few times too.” She also explained that she felt and still feels responsible for her mother and that she sometimes did not invite friends to come play to not overburden her mother. The daughter expressed that she would have appreciated guidance, fo example, by the general practitioner, during her childhood. She received psychological counseling in her 20s. Right now, the daughter is doing well.

In summary, this is the first time a child is old enough to recognize the emotional challenges of being born after maternal LT. This might call for increased awareness and support. However, the outcomes of this family cannot be generalized, for example, because the frequency of hospitalizations and the experienced anxiety will differ between families. This case shows that the experiences within a family can be different from each other. Therefore, we believe future studies should focus on long-term outcomes of mother, father, and child, to understand if the quality of life of more families is impacted by pregnancy after LT, and if additional support is needed. As a first step, we recommend examining experiences of more families in a qualitative study with individual interviews.

## ACKNOWLEDGMENTS

The authors gratefully acknowledge the kind help they received in the writing of this report through the extensive interviews with the patient, her husband, and her daughter.
